# Association of cytotoxic T-lymphocyte-associated protein 4 polymorphisms with recurrent pregnancy loss: A case-control study

**DOI:** 10.18502/ijrm.v21i1.12664

**Published:** 2023-02-08

**Authors:** Fereshteh Vaziri Nezamdoust, Hossein Hadinedoushan, Nasrin Ghasemi

**Affiliations:** ^1^Reproductive Immunology Research Center, Shahid Sadoughi University of Medical Sciences, Yazd, Iran.; ^2^Abortion Research Center, Yazd Reproductive Sciences Institute, Shahid Sadoughi University of Medical Sciences, Yazd, Iran.

**Keywords:** Recurrent pregnancy loss, Single nucleotide polymorphisms, CTLA-4 gene.

## Abstract

**Background:**

A large proportion of cases of recurrent pregnancy loss (RPL) are associated with immunological factors.

**Objective:**

This study investigated the association between single nucleotide polymorphisms of cytotoxic T-lymphocyte-associated protein (*CTLA)-4* gene in women with a history of RPL compared to healthy women.

**Materials and Methods:**

A case-control study was performed on 2 groups consisting of 120 healthy women with no history of abortion and at least one delivery (control) and 120 women with a history of 2 or more primary RPLs (case). In addition, 5 mL of peripheral blood sample was taken from all subjects. The frequencies of CTLA-4 rs3087243 and rs231775 polymorphisms were assayed by restriction fragment length polymorphism polymerase chain reaction and rs5742909 using the high-resolution melting real-time polymerase chain reaction method.

**Results:**

The mean age of the women in the control and RPL groups were 30.03 ± 4.23 (range 21-37), and 28.64 ± 3.61 yr (range 20-35), respectively. Pregnancy loss numbers ranged between 2-6 in women with a history of RPL, and between 1 and 4 in the successful pregnancy group. Statistical analysis showed a significant difference between the genotypes of GG and AG in the 2 groups in rs3087243 polymorphism (OR 1.00 for GG genotype and OR 2.87 for AG genotype, p = 0.0043). No significant difference was observed in the genotype frequencies of rs231775 and rs5742909 polymorphisms, of the 2 groups (p = 0.37, and p = 0.095), respectively.

**Conclusion:**

Our findings indicated that CTLA-4 polymorphism, rs3087243, might be associated with a risk of RPL in Iranian women.

## 1. Introduction

Approximately, 10-15% of women of childbearing age are not able to have children for unknown reasons (1). Researchers have been trying to determine the relationship between reproductive failure including recurrent abortion and infertility with an immunological background (2, 3). Abortion is one of the most common problems; and about 10-15% of pregnancies lead to abortion (4, 5). Abortion is the termination of pregnancy in any case before the embryo evolves into a fetus sufficiently to survive (6-9). Recurrent pregnancy loss (RPL) is defined as at least 2 consecutive pregnancy losses before 20 wk of gestation. Although several etiologic factors have been described, the cause of RPL cannot be determined clearly in almost 50% of the cases (10). However, only a few studies have revealed links between specific genetic variants and RPL's origin. Many studies have previous study has shown that immune factors play an important role in RPL development, such as the imbalance of T-helper (Th) 1 and Th2 are associated with the outcomes of pregnancy (11). Out of many antigenic systems cytotoxic T-lymphocyte- associated protein (*CTLA*)-4 is considered one of the most important so far. This protein is a homodimer leading to changing signal transduction and producing cytokines. One of these cytokines is tumor necrosis factor-beta which is a cytokine immune modulator during pregnancy. This marker is expressed in activated T-cells which regulates the inhibition and tolerance of this cells. Displacement of this factor on the cell surface after T-cell activation results in the transduction of a negative signal and T-cell inactivity (12). During normal pregnancy, the immune response between mother and fetus is minimized that caused by the inhibition of infiltration of lymphocytes. *CTLA-4* gene expression in fibroblasts and placental cells increases throughout pregnancy resulting in intolerance during this period (13). Some studies could detect *CTLA-4* proteins in embryonic fibroblast cells indicating that *CTLA-4* abnormal or low expression in paired fibroblast cells is associated with repeated abortions (13-15). To identify dominant immunological mechanisms during pregnancy, extensive studies have been done on the gene expression pattern in maternal and fetal immune responses, which indicate that *CTLA-4* is one of the important genes in this process.

The human *CTLA-4* gene is located on band q33 of chromosome 2. This gene has 4 exons, and several single nucleotide polymorphisms (SNPs) have been reported. The rs5742909 is located in the promoter site of the gene and plays a role in the rate of incidence of *CTLA-4* molecule. The rs4553808 is the linking site of the transcription factor of C/EBPR and affects the differentiation, proliferation, and apoptosis of cells. These SNPs are important as they modify the immune response of the host by affecting *CTLA-4* gene expression, protein production, *CTLA-4* link, and CD80 ligand (16, 17).

Despite the important role of *CTLA-4* in pregnancy, little information has been reported about the gene polymorphism of *CTLA-4* with RPL. In this study, we surveyed the genetic association of *CTLA-4* rs3087243, rs231775, and rs5742909 polymorphisms RPL group in women in the Iranian population.

## 2. Material and Methods

The target population included 120 women with RPL as the case group and 120 women without RPL as the control group referred to Yazd Reproductive Sciences Institute, Yazd, Iran that were in the reproductive age of 20-35 yr. The size of the sample depends mainly on the frequency of the least frequent allele or genotype. A sample size of 100 individuals will retain genotypes (alleles) that arise at frequencies of 5% (18, 19). However, considering the formula of comparing 2 proportions and similar studies the appropriate sample size was calculated as 120. The average effect size was calculated as 0.3 due to the difference in the reported results (14, 20, 21). The confidence level and power of the test are considered respectively 0.95, α is 0.05, and 0.8. In this regard, the women with at least 2 or more abortions and no history of viable birth were examined. In addition, women with endocrine disorders- over or lower production of hormones- such as diabetes mellitus and thyroid disorders, chromosomal aberrations, hyperprolactinemia, antiphospholipid syndrome, thrombotic disorders, anatomical disorders of the genital system, and urinary tract infections were excluded from the study. After conducting clinical examinations and paraclinical records of women, the uncertainty of the cause of their abortion was confirmed.

The control group included women having no history of abortion, stillbirth, infertility, and having at least one healthy child without using any assisted reproductive technology. Patient and control groups were matched for age, and 5 mL of blood was taken from samples and stored in an ethylene diamine tetraacetic acid (EDTA)-coated tube at the time of enrolment.

### Experimental materials

Materials for high-resolution melting analysis (HRM-PCR): Solis bioDyne HRM-real time PCR kit that master mix containing Mgcl2, Buffer 10x, dNTP, fluorescent dye ogrerin plus extracted DNA, primer, sterile deionized water. All the materials were provided by Merck (Darmstadt, Germany). Materials for Salting out DNA extraction: Red cell lysis buffer (containing NH4Cl- KHCO3 -EDTA -NaOH), nucleus of the cell lysis buffer (containing Tris - EDTA- NaCl- HCl, PH), Proteinase k, Sodium dodecyl sulfate, Sodium chloride (NaCl), ethanol 100%, ethanol 70%, TE solution (Tris-EDTA), NaoH solution. These materials were provided by Merck (Darmstadt, Germany), too.

### Genetic analysis

The polymorphisms of rs3087243 and rs231775 *CTLA-4* were analyzed by the polymerase chain reaction (PCR)-restriction fragment length polymorphism (RFLP) technique. DNA was extracted from whole blood samples (120 cases and 120 controls) using AceuPrep Genomic DNA extraction kit according to the manufacturer's instructions. Obtained DNA was stored at -20 C until used. Sequences containing the SNPs rs3087243 and rs231775 were located in gene bank, and primers were designed by PRIMER3 to amplify the appropriate DNA fragments. For SNP rs3087243 and SNP rs231775, primer sequences are shown in table I.

Each PCR reaction was carried out in a 25 μL tube containing, 12.5 μL master mix, 3 μL of DNA, 2 μL of each of the forward and reverse primers and 7.5 μL distilled water. The PCR cycling consisted of an initial denaturation at 94 C for 5 min, followed by 35 cycles of denaturation for 1 min at 94 C, annealing for 45 sec at 57 C, and extension for 45 sec at 72 C by RT-PCR device (Applied Biosystems, ABI, Foster City, CA, USA). Electrophoresis of the PCR products was performed using 1.5% agarose gel stained with a green viewer. The PCR product size was digested by restriction enzymes, N1aIII for rs3087243 and Apek1 for rs231775 according to the instruction given by the manufacturer (Fermentas, CA). All the gels were imaged using an E-gel imager; 10% of the samples were randomly selected, the assays were repeated, and the results were 100% consistent with the initial analyses. We used a high-resolution melting analysis for rs5742909 *CTLA-4* in 120 samples as cases and 120 samples as controls. The reaction was performed in a 20-μL tube containing 2 μL of DNA, 4 μL master mix (5x HOT FIREPol Eva GREEN HRM Mix), 12 μL distilled water, and 2 μL of each of the forward and reverse primers. The primer was designed by PRIMER3 (Table I).

The PCR cycling consisted of an initial denaturation at 95 C foFr 15 min, followed by 40 cycles of denaturation for 15s at 95 C, annealing for 20 s at 63 C and extension for 20 s at 72 C (Rotor-Gene 6000 Corbett Research, Sydney, Australia). The analyses were done by Rotor-Gene Q2.3.

**Table 1:** Primers used for detection of rs3087243, rs231775, rs5742909 in Cytotoxic T-lymphocyte-associated protein gene


**RS number**	**Primer sequence**
	F: 5'TTCCATGAAAATGCAACAACA 3'
**rs3087243**	R: 5'AAGTGGAAACCAAATGTGCTG 3'
		F: 5'TGGTTAAGGATGCCCAAAG 3'
**rs231775**	R: 5'CACTGCCTTTGACTGCTGAA 3'
		F: 5'GGGATTTAGGAGGACCCTTG 3'
**rs5742909**	R: 5' AGCCGTGGGTTTAGCTGTTA 3'

### Ethical considerations

The protocol was approved by the Ethics Committee of Shahid Sadoughi University of Medical Sciences, Yazd, Iran (Code: IR.SSU.MEDICINE REC.1394.462). Informed written consent was obtained from all participants.

### Statistical analysis

The statistical analyses were conducted using Statistical Product and Service Solutions (SPSS) 16 (SPSS Inc., Chicago, IL, USA). SNPs were evaluated for deviation from Hardy-Weinberg Equilibrium for both RPL and the control groups. The Chi-square test was used to evaluate the differences between the genotype frequency in RPL women and the control group. P ≤ 0.05 was considered statistically significant.

## 3. Results

**Figure 1:**
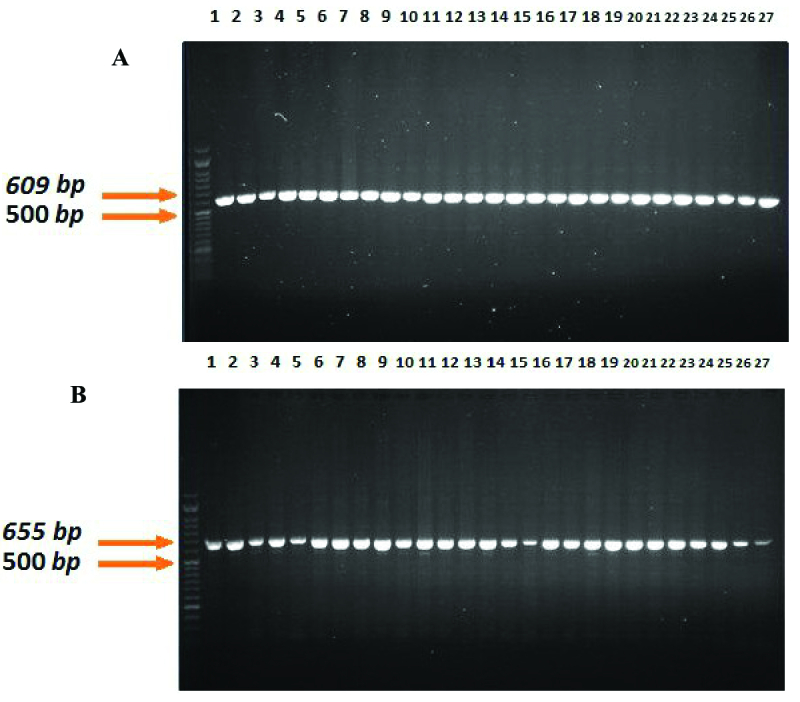
The PCR result of rs3087243, before (A) and after (B) enzyme restriction.

**Figure 2:**
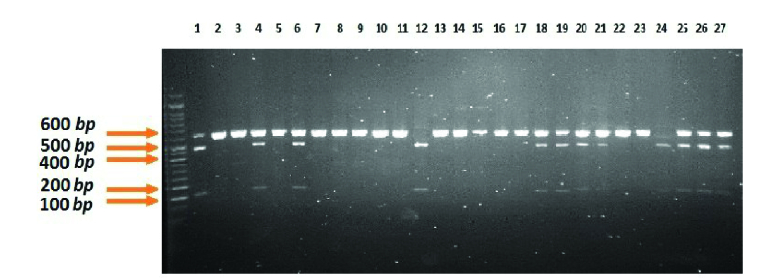
The RFLP results of rs231775. AA: 609 bp. AG: 146bp, 463bp, 609bp. GG: 146bp, 463bp.

**Figure 3:**
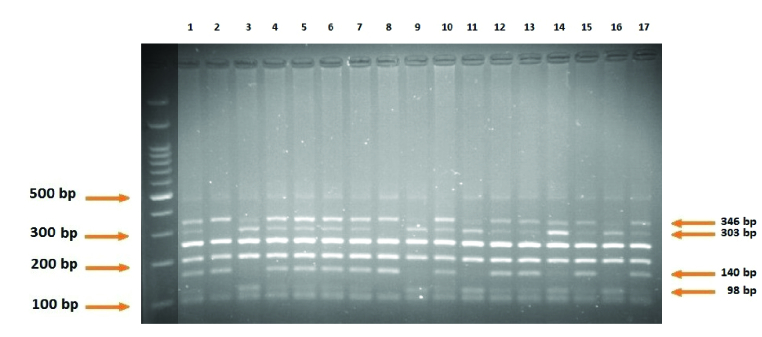
The RFLP results of rs3087243. AA: 98 bp, 303bp, 346 bp (3-9-11-14). AG: 98bp, 140bp, 303bp, 346bp. GG: 140bp, 346bp (2-8).

The mean and range of age are displayed in table II for both RPL and control groups. No significant difference was observed in the age of women between the 2 groups. For verification of correctness of experimental, gel's picture before and after enzyme restriction by PCR and RFLP methods are shown in figures 1-3 respectively.

All genotypes of the polymorphism were demonstrated by the Hardy-Weinberg Equilibrium. The frequencies of genotypes in the RPL and control groups were shown in table III. In addition, the allele frequencies of this polymorphism (A and G) for both groups were displayed. Regarding codominant inheritance model analysis, a statistically significant difference in the genotype distribution of the rs3087243 was seen between the patients and control subjects (p = 0.0043, OR 1.00 for GG genotype, OR 2.87 for AG genotype, and OR 1.72 for AA genotype). A statistically significant difference was found in genotype frequencies of rs3087243 polymorphism between the RPL and the control groups (p = 0.02).

For rs231775, the frequencies of genotypes in the RPL group were AG (22.5%), AA (51.7 %), and GG (25.8 %). Furthermore, they were AG (25%), AA (56.7%), and GG (18.3 %) in the control group (table III). The genotype frequencies and A and G alleles in the 2 groups were not significantly different (p = 0.37 and p = 0.17 respectively, OR 1.00 for AA genotype, OR 1.01 for AG genotype, and OR 0.65 for GG genotype).

The allele frequencies of this polymorphism were T (31%), C (69%) in the RPL group, and T (22%), C (78%) in the control group. The frequencies of genotypes frequencies and T and C alleles in the 2 groups were not significantly different (p = 0.095 and p = 0.06, respectively Table III).

**Table 2:** Characteristics of the recurrent pregnancy loss and the control group


**Variables**	**Recurrent pregnancy loss**	**Control**
**Age (yr)**	28.64 ± 3.61 (20-35)	30.03 ± 4.23 (21-37)
**No. of abortion**	3.8 ± 1.6 (2-6)	0
**No. of successful pregnancy **	0	2.24 ± 0.98 (1-4)
Data presented as Mean ± SD (range)		

**Table 3:** Frequency of single nucleotide polymorphism of rs3087243, rs231775, and rs5742909 between groups


		**Genotype**	**Case**	**Control**	**Odds ratio (95% CI)**	**P-value**
**rs3087243**						
	G/G	38 (31.7)	19 (15.8)	1.00	
	A/G	53 (44.2)	76 (63.4)	2.87 (1.49-5.51)	
	**Codominant**	A/A	29 (24.1)	25 (20.8)	1.72 (0.80-3.72)	0.0043
		G/G	38 (31.7%)	19 (15.8)	1.00 (Reference)		
		**Dominant**	A/G-A/A	82 (68.3)	101 (84.2)		2.46 (1.32-4.59)	0.0037
	G/G-A/G		91 (75.8)	95 (79.2)	1.00 (Reference)			
	**Recessive**	A/A	29 (24.2)	25 (20.8)	0.83 (0.45-1.52)	0.54
		G/G-A/A	67 (55.8)	44 (36.7)	1.00 (Reference)		
	**Over dominant**	A/G	53 (44.2)	76 (63.3)	2.18 (1.30-3.66)	0.002
		**Log-additive**	—	—	—		1.31 (0.90-1.91)	0.15
	G	129 (54)	114 (48)	1.00 (Reference)	
	**Alleles**	A	111 (46)	126 (52)	1.28 (0.90-1.82)	0.02
**rs231775**						
	A/A	62 (51.7)	68 (56.7)	1.00	
	A/G	27 (22.5)	30 (25)	1.01 (0.54-1.89)	
	**Codominant**	G/G	31 (25.8)	22 (18.3)	0.65 (0.34-1.23)	0.37
		A/A	62 (51.7)	68 (56.7)	1.00 (Reference)		
		**Dominant**	A/G-G/G	58 (48.3)	52 (43.3)		0.82 (0.49-1.36)	0.44
	A/A-A/G		89 (74.2)	98 (81.7)	1.00 (Reference)			
	**Recessive**	G/G	31 (25.8)	22 (18.3)	0.64 (0.35-1.19)	0.16
		A/A-G/G	93 (77.5)	90 (75)	1.00 (Reference)		
	**Over dominant**	A/G	27 (22.5)	30 (25)	1.15 (0.63-2.08)	0.65
		**Log-additive**	—	—	—		0.83 (0.60-1.13)	0.23
	A	151 (63)	166 (69)	1.00 (Reference)	
	**Alleles**	G	89 (37)	74 (31)	2.08 (1.37-3.17)	0.17
**rs5742909**						
	C/C	67 (55.8)	77 (64.2)	1.00	
	C/T	32 (26.7)	33 (27.5)	0.90 (0.50-1.61)	
	**Codominant**	T/T	21 (17.5)	10 (8.3)	0.41 (0.18-0.94)	0.095
		C/C	67 (55.8)	77 (64.2)	1.00 (Reference)		
		**Dominant**	C/T-T/T	53 (44.2)	43 (35.8)		0.71 (0.42-1.19)	0.19
**rs5742909**								
	C/C-C/T	99 (82.5)	110 (91.7)	1.00 (Reference)	
	**Recessive**	T/T	21 (17.5)	10 (8.3)	0.43 (0.19-0.95)	0.033
		C/C-T/T	88 (73.3)	87 (72.5)	1.00 (Reference)		
	**Over dominant**	C/T	32 (26.7)	33 (27.5)	1.04 (0.59-1.84)	0.88
		**Log-additive**	—	—	—		0.71 (0.49-1.01)	0.056
	C	166 (69)	187 (78)	1.00 (Reference)	
	**Alleles**	T	74 (31)	53 (22)	1.573 (1.04- 2.35)	0.06
Data presented as n (%). Chi-square test						

## 4. Discussion

Some polymorphisms of *CTLA-4* could be recognized as one of the factors predisposing to RPL. The fetus is a semi-allograft that is not rejected by the maternal immune system during pregnancy. The tolerance created at the fetus-mother contact site during pregnancy is vital for maintaining the fetus and lack of its rejection by the maternal immune system (16). The role of immunologic factors in RPL has been extensively investigated. In our previous studies, we indicated the role of imbalance of subgroups of Th cells and also the role of some gene polymorphisms of molecules that contribute to this imbalance (22, 23). The *CTLA-4* molecule that is homologous with the CD28 co-stimulator molecule appears in their membrane after T-cell activation. Unlike CD28 molecules, this molecule plays a down-regulation role in activating the T-cells (24). The results of a study showed that the rate of incidence of *CTLA-4* in the clones of Th2 cells was greater than Th1 cells and that this molecule contributes to the inhibition of the production of cytokines secreted by Th1 and Th2 cells (25). The aberrant expression of co-stimulatory molecules at the mother-fetus contact site may affect the abortion. A study demonstrated that the expression of *CTLA-4* molecules in deciduas in women with a history of RPL was reduced compared to controls with normal pregnancy (26). An increased number of *CTLA-4* ${}^{+}$ cells in the endometrial wall of women with repeated miscarriages has been reported. This increase shows a positive proportion to an increased number of FOXP3 ${}^{+}$ indicating the synergistic effects of *CTLA-4* ${}^{+}$ and FOXP3 ${}^{+}$ cells in the downregulation of inflammation in the endometrium of women with recurrent implantation failure (27). Given the possible role of this molecule and the related polymorphisms of its gene in the rate of activity and performance of this molecule in RPL, in this study, the SNPs of the *CTLA-4* gene including rs3087243, rs231775, and rs5742909 were compared in women with RPL and healthy controls in the Iranian women. The results revealed that the difference was significant in rs3087243 genotypes frequencies, though the difference was not significant in other genotypes under study. The correlation between some SNPs of the *CTLA-4* gene and the risk of RPL has been explored in different populations. The findings of some studies suggest that the correlation between *CTLA-4* gene polymorphisms and RPL varies among different populations such as a study investigated 133 women with a history of RPL and 146 healthy women as controls for polymorphisms of rs4553808, rs5742909, rs231775, and rs733618. The AA genotype was more frequent for rs231775 in the control group compared to the RPL group. For rs5742909, the CT genotype frequency was insignificantly higher in controls compared to the abortion group. The T allele was significantly more frequent in controls indicating the association between this allele and successful pregnancy and reduced risk of RPL (15). The correlation between frequencies of *CTLA-4* gene polymorphisms and the incidence of RPL in the Chinese Han population has been examined. The results of this study demonstrated that the G allele in rs231775 exerts a protective effect in the incidence of RPL compared to the A allele while allele A in rs3087243 showed a protective role in women with RPL compared to controls (28). In another study in the Chinese population, the frequencies of alleles and genotypes in rs231775 were examined in women with unexplained RPL and controls. The results suggested that the alleles and genotypes were significantly different between the 2 groups so that A/G polymorphism was associated with the immunopathogenesis of RPL (12). The association between *CTLA-4* gene polymorphisms and other pregnancy problems has been also investigated in different populations. The results of the 2 studies demonstrated no significant association between different genotypes and alleles of this gene in women afflicted with preeclampsia compared to controls (29, 30). Misra et al. explored the correlation between several polymorphisms of the *CTLA-4* gene and the serum levels and susceptibility of affliction with idiopathic recurrent miscarriage in the Indian population. They found that the A and G alleles in rs231775 and rs3087243 were different in the RPL group compared to controls; yet, the difference was not significant for other polymorphisms under study including T and C alleles in rs3087243 (31). Another study explored the correlation between the rs231775 polymorphism of the *CTLA-4* gene and repeated spontaneous abortions among Iranian Azari women. The results indicated no significant correlation between “frequencies of genotypes and the related alleles” and “susceptibility of affliction with abortion” (32). As mentioned, the correlation between various polymorphisms of the *CTLA-4* gene and susceptibility to affliction with RPL has been studied in different populations. The results of these studies were consistent with our findings, though some of them contradict our results. This may be due to the different prevalence of genotypes in various populations or differences in race. Also, the type and number of samples may contribute to this disparity. However, the findings of another study indicated a correlation between the G allele in rs231775 and susceptibility of affliction with pre-eclampsia (33). A study on infertile Brazilian women with or without endometriosis suggested that the rs231775 polymorphism plays no role in the pathogenesis of endometriosis (34). Furthermore, the results of a study on women with polycystic ovary syndrome indicated a significant difference in genotype and allele frequencies of rs733618 in these women compared to controls so that the T allele was significantly higher in controls; yet, the 2 groups were not significantly different in rs231775 (35). Additionally, the correlation between *CTLA-*4 polymorphisms and susceptibility of affliction with autoimmune disorders has been studied leading to controversial results. Although no correlation was found between rs231775 and affliction with systemic lupus erythematosus in Indian (36), such a correlation was found among the Japanese population (37). Moreover, the correlation between rs231775 polymorphism and susceptibility of affliction with rheumatoid arthritis was examined in the Chinese population. The results revealed that the genotype and allele frequencies were significantly different between the RPL and the control groups so that allele A was associated with an increased risk of affliction with rheumatoid arthritis (38). Finally, the 3 genotypes of the *CTLA-4* gene were compared between individuals with autoimmune hepatitis and controls. The results suggested a significant difference in rs231775; yet, the difference was not significant about other alleles (39).

## 5. Conclusion

The polymorphisms of *CTLA-4* may affect the role of this molecule in the regulation of immune responses, especially the mother-to-fetus immune response leading to the incidence of RPL. Our results showed that the rs3087243 polymorphism of the *CTLA-4* molecule serves as one of the factors predisposing to RPL. Future studies on larger populations using various samples are required to focus on determining the association between *CTLA-4* polymorphisms and affliction with RPL.

##  Conflict of Interest 

The authors declare that there is no conflict of interest.
